# Supplementary material for: World Trade Center responders in their own words: predicting PTSD symptom trajectories with AI-based language analyses of interviews
Source: Psychol Med. 2021 Jun 22;53(3):918–26. doi: 10.1017/S0033291721002294 (PMC8692489; doi:10.1017/S0033291721002294)
Supplement: Supplementary file 1 [file S0033291721002294sup001.docx]

**Table S1**. Analysis on suppression effects of confound variables for PCL trajectories

|  | **Interview**  **Language Features** | **PTSD Symptoms Trajectories** (Future Change) | | | | | | |
| --- | --- | --- | --- | --- | --- | --- | --- | --- |
|  |  | ***r***  [95% CI] | $\boldsymbol{\beta}$ **(adjusted for each confound variable)**  [95% CI] | | | | | |
|  |  | Unadjusted | Occupation | Gender | Days since 9/11 | Interview PCL | Age | Adjusted^1^ |
| **Psychological**  **Traits** | Anxiety | 0.16 [-0.07, 0.37] | 0.17  [-0.06, 0.38] | 0.21  [-0.02, 0.41] | 0.14  [-0.09, 0.35] | 0.25  [0.03, 0.45] | 0.21  [-0.02, 0.41] | 0.30*  [0.07, 0.50] |
|  | Depression | 0.00  [-0.23, 0.22] | 0.00  [-0.22, 0.23] | 0.05  [-0.18, 0.27] | 0.00  [-0.23, 0.23] | 0.13  [-0.10, 0.35] | 0.03  [-0.20, 0.25] | 0.16  [-0.07, 0.37] |
|  | Neuroticism | 0.07  [0.29, -0.16] | 0.07  [-0.16, 0.29] | 0.12  [-0.11, 0.34] | 0.06  [-0.17, 0.28] | 0.18  [-0.05, 0.39] | 0.09  [-0.14, 0.31] | 0.20  [-0.03, 0.40] |
|  | Extraversion | 0.17  [-0.06, 0.38] | 0.17  [-0.06, 0.38] | 0.21  [-0.01, 0.42] | 0.17  [-0.06, 0.38] | 0.15  [-0.09, 0.36] | 0.18  [-0.05, 0.39] | 0.18  [-0.05, 0.39] |
| **Linguistic**  **Style** | First-Person Singular | 0.00  [-0.23, 0.23] | -0.01  [-0.24, 0.22] | 0.01  [-0.22, 0.24] | 0.04  [-0.19, 0.26] | 0.10  [-0.13, 0.32] | 0.02  [-0.21, 0.25] | 0.13  [-0.09, 0.32] |
|  | First-Person Plural | -0.36*  [-0.54, -0.14] | -0.36*  [-0.54, -0.14] | -0.33*  [-0.52, -0.11] | -0.34*  [-0.53, -0.13] | -0.37*  [-0.55, -0.16] | -0.37*  [-0.55, -0.16] | -0.36*  [-0.54, -0.15] |
|  | Articles | -0.16  [-0.37, 0.07] | -0.19  [-0.40, 0.04] | -0.19  [-0.40, 0.04] | -0.13  [-0.35, 0.10] | -0.19  [-0.40, 0.04] | -0.19  [-0.40, 0.04] | -0.23  [-0.43, -0.00] |
|  | AVG Word Length | -0.36*  [-0.54, -0.14] | -0.37*  [0.55, -0.15] | -0.35*  [-0.53, -0.13] | -0.35*  [-0.54, -0.14] | -0.34*  [-0.53, -0.13] | -0.36*  [-0.54, -0.14] | -0.35*  [-0.53, -0.13] |
|  | Word Count | 0.06  [-0.17, 0.28] | 0.07  [-0.16, 0.29] | 0.10  [-0.13, 0.32] | 0.01  [-0.22,0.23] | 0.13  [-0.10, 0.35] | 0.09  [-0.14, 0.31] | 0.14  [-0.09, 0.46] |

*p<0.05, Benjamini-Hochberg (False-Discovery Rate) corrected.

^1^Adjusted for occupation, gender, days since 9/11, Interview PCL, and age

**Table S2**. Effects of marital status as a confound variable for cross-sectional interview PCL scores

|  | **Interview**  **Language Features** | **PTSD Symptoms** (concurrent, cross-sectional) | | | |
| --- | --- | --- | --- | --- | --- |
|  |  | ***r***  [95% CI] | $\boldsymbol{\beta}$ **(adjusted for each confound variable)**  [95% CI] | | |
|  |  | Unadjusted | Marital Status | Adjusted^1^ | Adjusted^2^ |
| **Psychological**  **Traits** | Anxiety | 0.26  [0.03, 0.46] | 0.25  [0.02, 0.45] | 0.20  [-0.03, 0.41] | 0.20  [-0.03, 0.41] |
|  | Depression | 0.38*  [0.16, 0.56] | 0.37*  [0.16, 0.55] | 0.32*  [0.10, 0.51] | 0.32*  [0.10, 0.51] |
|  | Neuroticism | 0.32*  [0.10, 0.51] | 0.31*  [0.09, 0.50] | 0.26  [0.03, 0.46] | 0.27  [0.04, 0.47] |
|  | Extraversion | -0.10  [-0.32, 0.13] | -0.09  [-0.31, 0.14] | -0.15  [-0.37, 0.08] | -0.14  [-0.36, 0.09] |
| **Linguistic**  **Style** | First-Person Singular | 0.31*  [0.09, 0.50] | 0.32*  [0.10, 0.51] | 0.31*  [0.09, 0.50] | 0.32*  [0.10, 0.51] |
|  | First-Person Plural | -0.05  [-0.28, 0.18] | -0.06  [-0.28, 0.17] | -0.10  [-0.32, 0.13] | -0.10  [-0.32, 0.13] |
|  | Articles | -0.09  [-0.31, 0.14] | -0.08  [-0.30, 0.15] | -0.06  [-0.28, 0.17] | -0.05  [-0.27, 0.18] |
|  | AVG Word Length | 0.05  [-0.18, 0.27] | 0.06  [-0.17, 0.28] | 0.03  [-0.20, 0.25] | 0.03  [-0.20, 0.25] |
|  | Word Count | 0.22  [-0.01, 0.43] | 0.20  [-0.03. 0.41] | 0.20  [-0.02, 0.41] | 0.19  [-0.04, 0.40] |

^1^Adjusted for age, gender, occupation, and days since 9/11.

^2^Adjusted for age, gender, occupation, days since 9/11, and marital status.

*p<0.05, Benjamini-Hochberg (False-Discovery Rate) corrected.

**Table S3**. Effects of marital status as a confound variable for PCL score future trajectories

|  | **Interview**  **Language Features** | **PTSD Symptoms Trajectories** (Future Change) | | | |
| --- | --- | --- | --- | --- | --- |
|  |  | ***r***  [95% CI] | $\boldsymbol{\beta}$ **(adjusted for confound variables)**  [95% CI] | | |
|  |  | Unadjusted | Adjusted^1^ | Adjusted^2^ | Adjusted^3^ |
| **Psychological**  **Traits** | Anxiety | 0.16  [-0.07, 0.37] | 0.25  [0.02, 0.45] | 0.30*  [0.07, 0.49] | 0.29*  [0.07, 0.49] |
|  | Depression | -0.00  [-0.23, 0.22] | 0.13  [-0.10, 0.35] | 0.16  [-0.06, 0.38] | 0.16  [-0.07, 0.37] |
|  | Neuroticism | 0.07  [0.29, -0.16] | 0.18  [-0.05, 0.39] | 0.20  [ -0.03, 0.40] | 0.20  [-0.03, 0.41] |
|  | Extraversion | 0.17  [-0.06, 0.38] | 0.15  [-0.08, 0.36] | 0.18  [-0.05, 0.39] | 0.18  [-0.05, 0.39] |
| **Linguistic**  **Style** | First-Person Singular | 0.00  [-0.23, 0.23] | 0.11  [-0.12, 0.33] | 0.13  [-0.10, 0.35] | 0.14  [-0.09, 0.36] |
|  | First-Person Plural | -0.36*  [-0.54, -0.14] | -0.38*  [-0.56, -0.16] | -0.36*  [-0.54, -0.15] | -0.37*  [-0.55, -0.15] |
|  | Articles | -0.16  [-0.37, 0.07] | -0.19  [-0.40, 0.04] | -0.23  [-0.43, 0.00] | -0.22  [-0.43, -0.00] |
|  | AVG Word Length | -0.36*  [-0.54, -0.14] | -0.34*  [-0.53, -0.12] | -0.35*  [-0.53, -0.13] | -0.35*  [-0.53, -0.13] |
|  | Word Count | 0.06  [-0.17, 0.28] | 0.13  [-0.10, 0.34] | 0.14  [-0.09, 0.36] | 0.14  [-0.09, 0.35] |

^1^ Adjusted for Marital Status and Interview PCL

^2^ Adjusted for age, gender, occupation, days since 9/11, and Interview PCL score.

^3^ Adjusted for age, gender, occupation, days since 9/11, interview PCL score, and marital status.

*p<0.05, Benjamini-Hochberg (False-Discovery Rate) corrected.

**Table S4.** *Cross-sectional association between language-based assessments and PHQ-9 Depression Scores*

|  | **Interview  Language Features** | **Depression (PHQ-9) Severity** | |
| --- | --- | --- | --- |
|  |  | ***r*** (direct correlation with symptom slope) | $\boldsymbol{\beta}$  (adjusted for age, gender, occupation, days since 9-11) |
| **Psychological**  **Traits** | Anxiety | 0.32* [0.10, 0.51] | 0.29 [0.07, 0.49] |
|  | Depression | 0.39* [0.18, 0.57] | 0.36* [0.14, 0.54] |
|  | Neuroticism | 0.39* [0.18, 0.57] | 0.34* [0.12, 0.53] |
|  | Extraversion | -0.11 [-0.33, 0.12] | -0.16 [-0.38, 0.07] |
| **Linguistic**  **Style** | First-Person Singular | 0.28* [0.06, 0.48] | 0.28 [0.05, 0.47] |
|  | First-Person Plural | -0.10 [-0.32, 0.13] | -0.12 [-0.33, 0.11] |
|  | Articles | -0.09 [-0.31, 0.14] | -0.06 [-0.28, 0.17] |
|  | AVG Word Length | 0.04 [-0.19, 0.26] | 0.04 [-0.19, 0.26] |
|  | Word Count | 0.21 [-0.02, 0.41] | 0.17 [-0.06, 0.38] |

*p<0.05, Benjamini-Hochberg (False-Discovery Rate) corrected.

**Table S5.** *Language features used by each type of language based assessments. All of the features and model scores were generated by DLATK.*

| **Language-based Assessments** | **Linguistic Features** | **Implementation Details** |
| --- | --- | --- |
| **Psychological**  **Traits** | - Relative frequencies of words and phrases - Binary indicators of words and phrases - 2,000 LDA topics introduced by Schwartz et al. (2013) | - Depression and anxiety language models used in our analysis were explored and validated in the paper (Schwartz et al., 2014). - Neuroticism and extraversion language models used in our analysis were explored and validated (Park et al., 2015). - All of the linguistic features in the ‘Linguistic Features' column on this row were extracted from oral history interviews and used as input to these state-of-the-art AI-based language models for the PTSD analysis we conducted in clinical settings. |
| **Linguistic Styles** | - Frequencies of words - Category scores were computed from relative frequencies of the words in each category | - Words from the oral history interviews were counted and mapped to the terms of LIWC 2015 categories (Pennebaker et al., 2015) or meta features, and then these were computed as linguistic style scores. - We selected language categories and meta features which were found to be correlated with PTSD or other related psychological variables (see ‘Function Word Lexicon Features’ and ‘Language Meta Features’ of our method section). |
